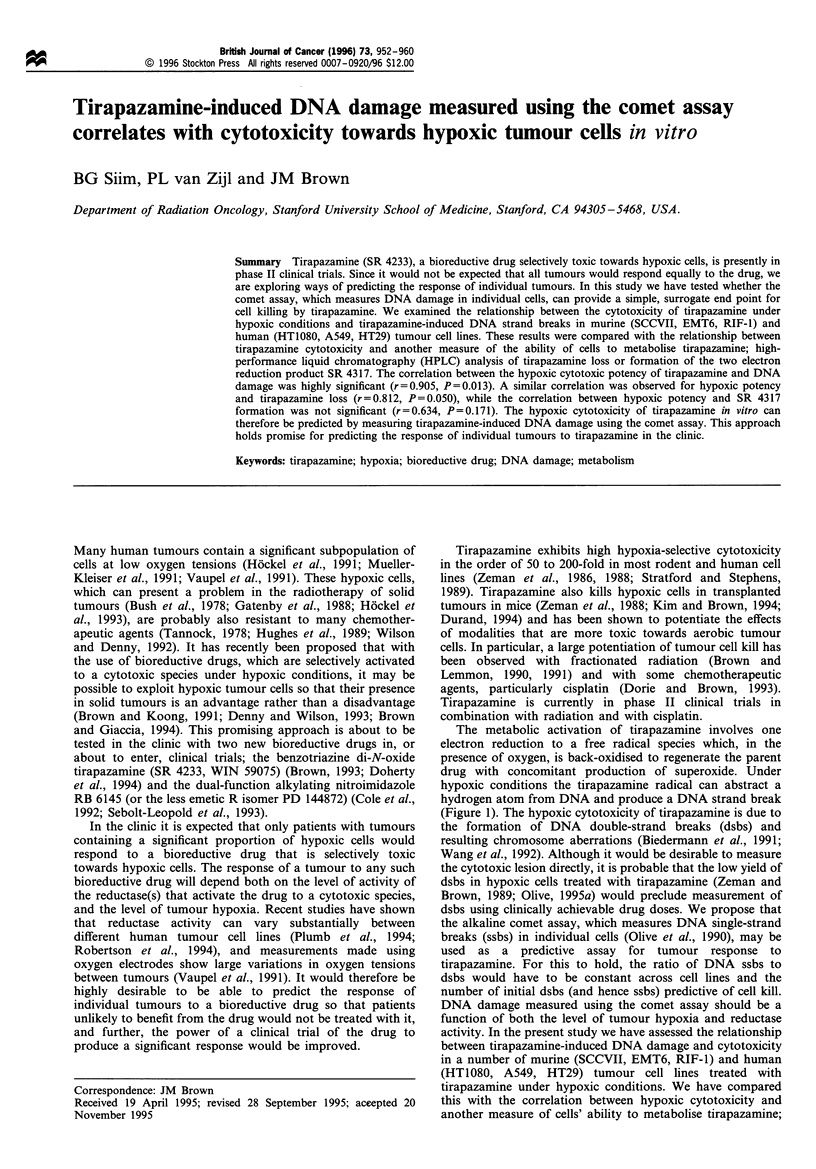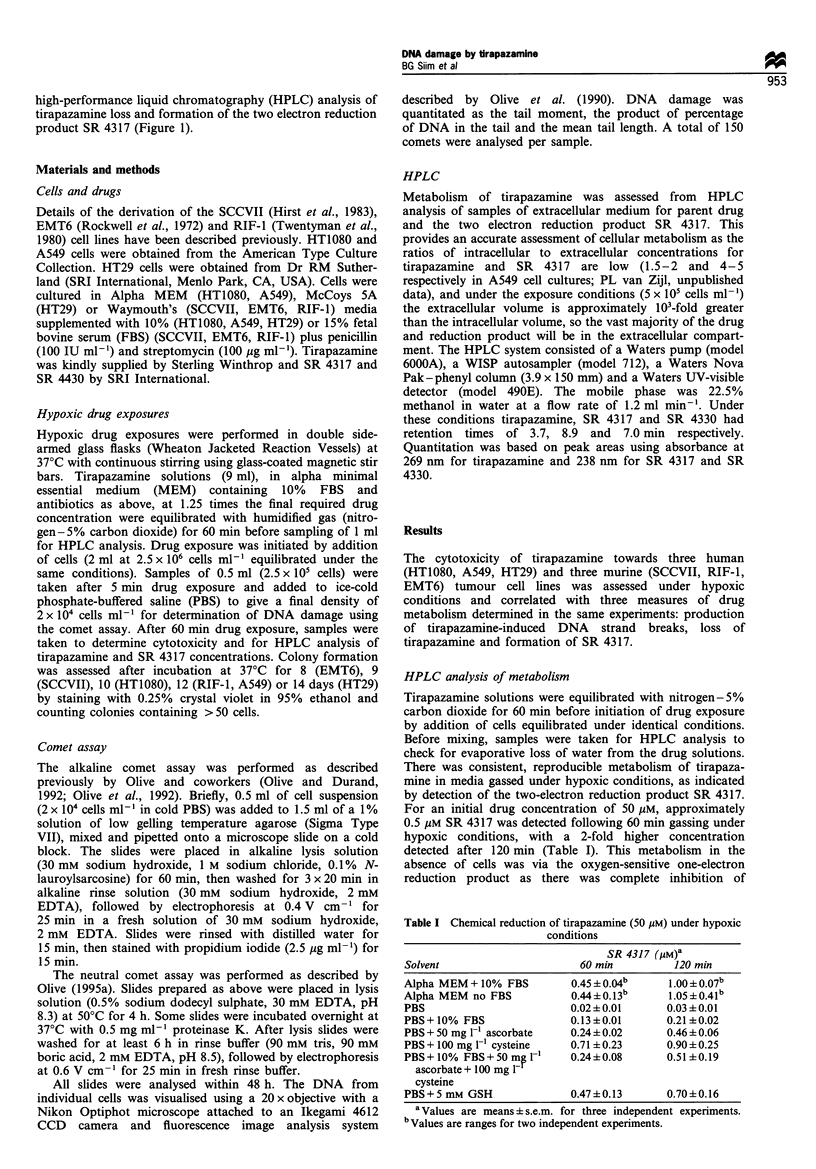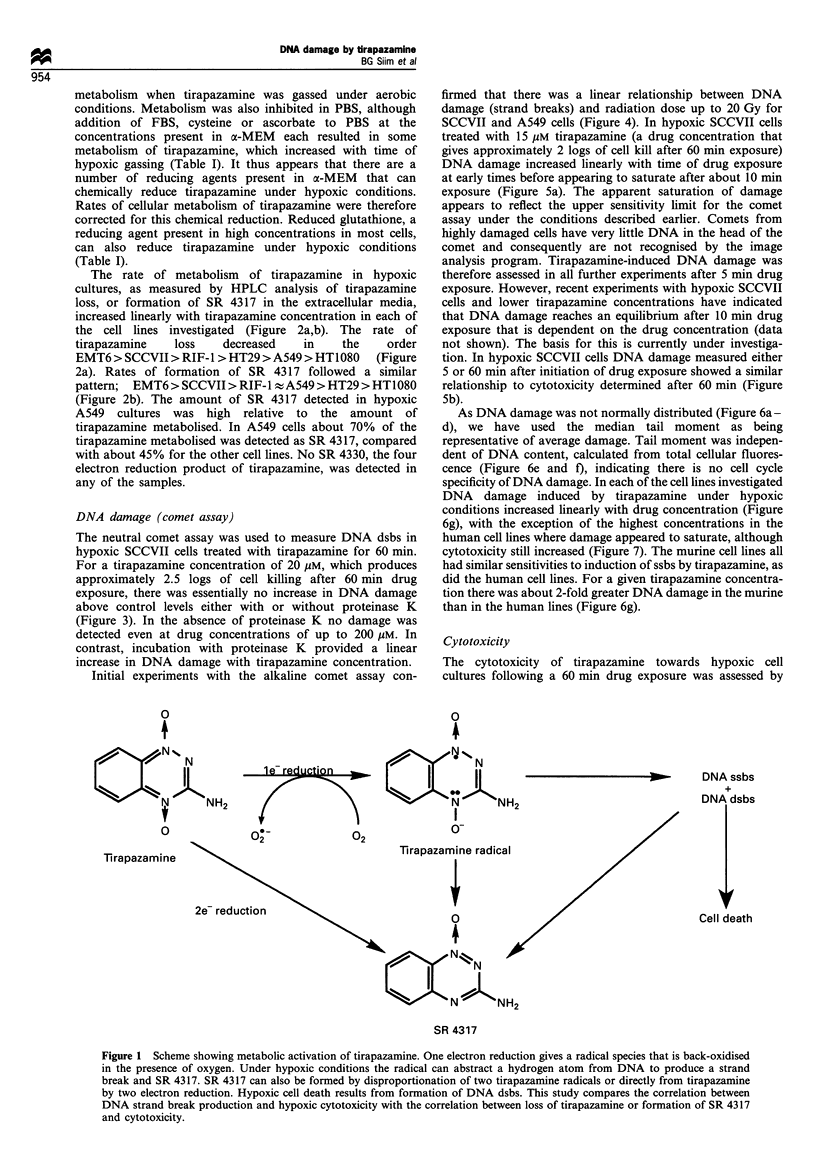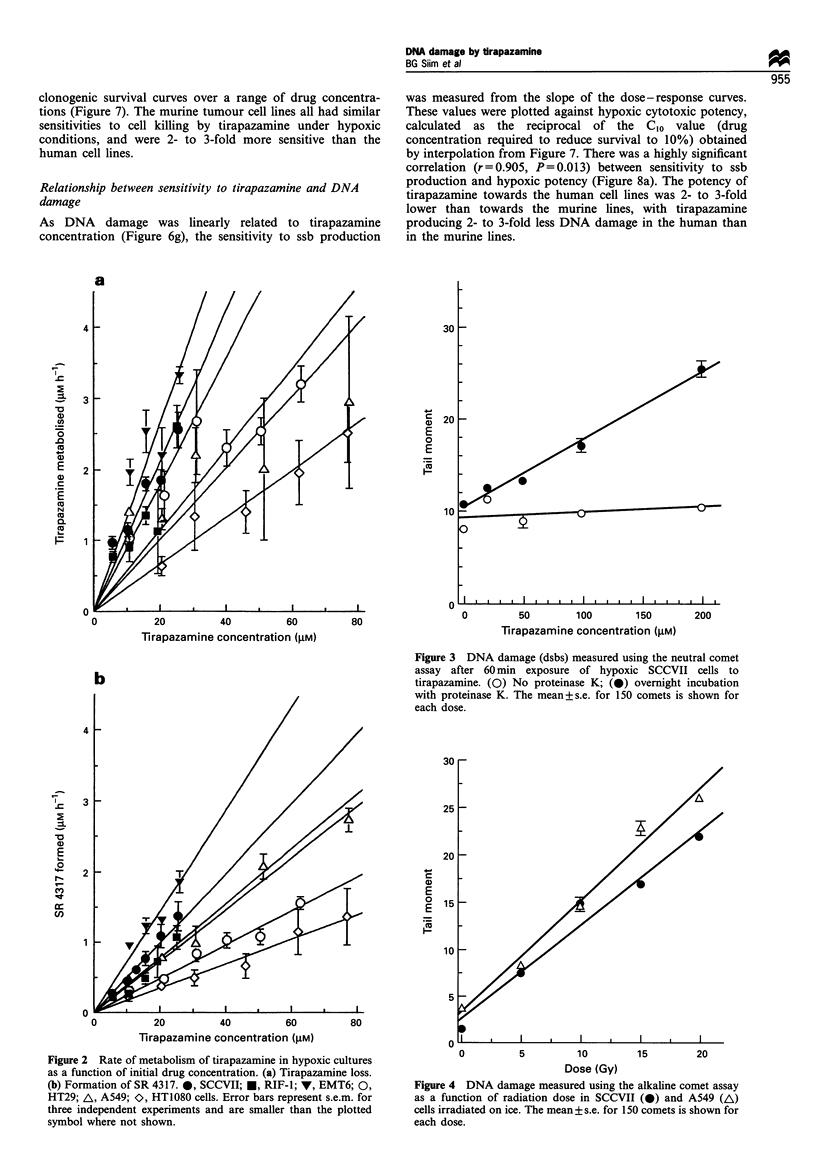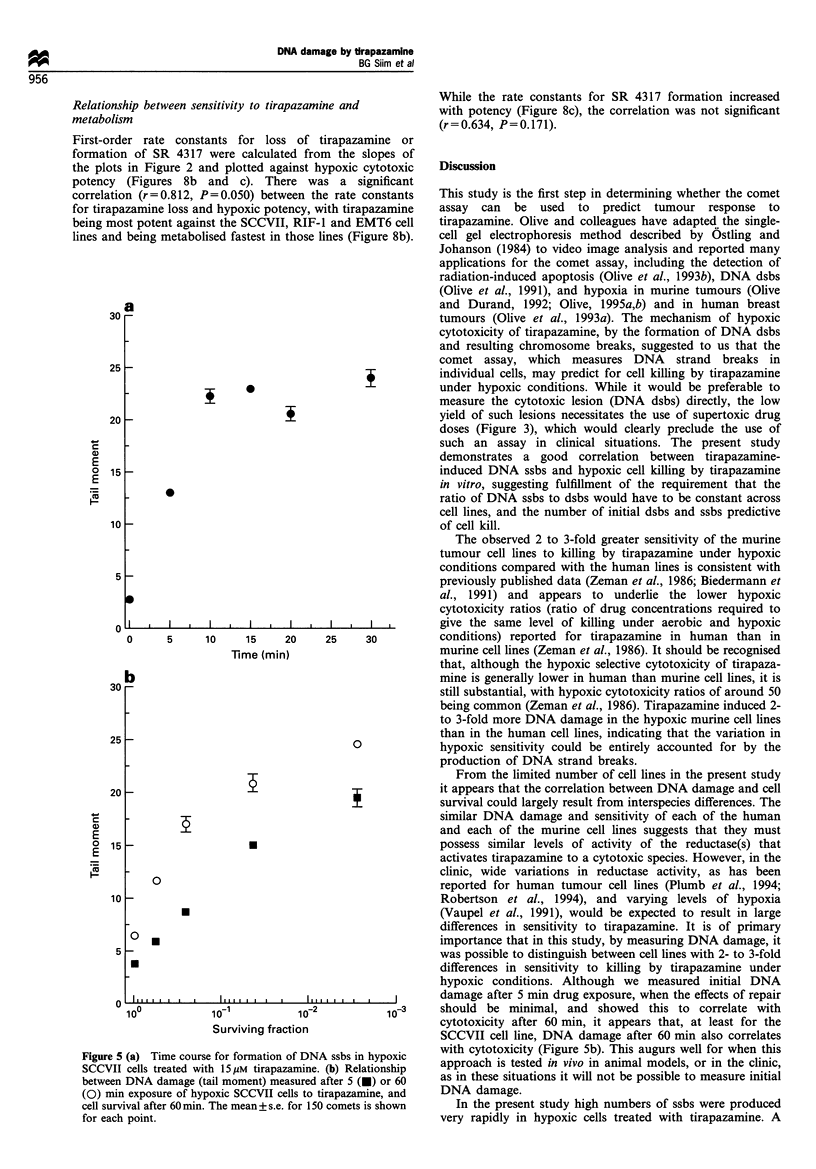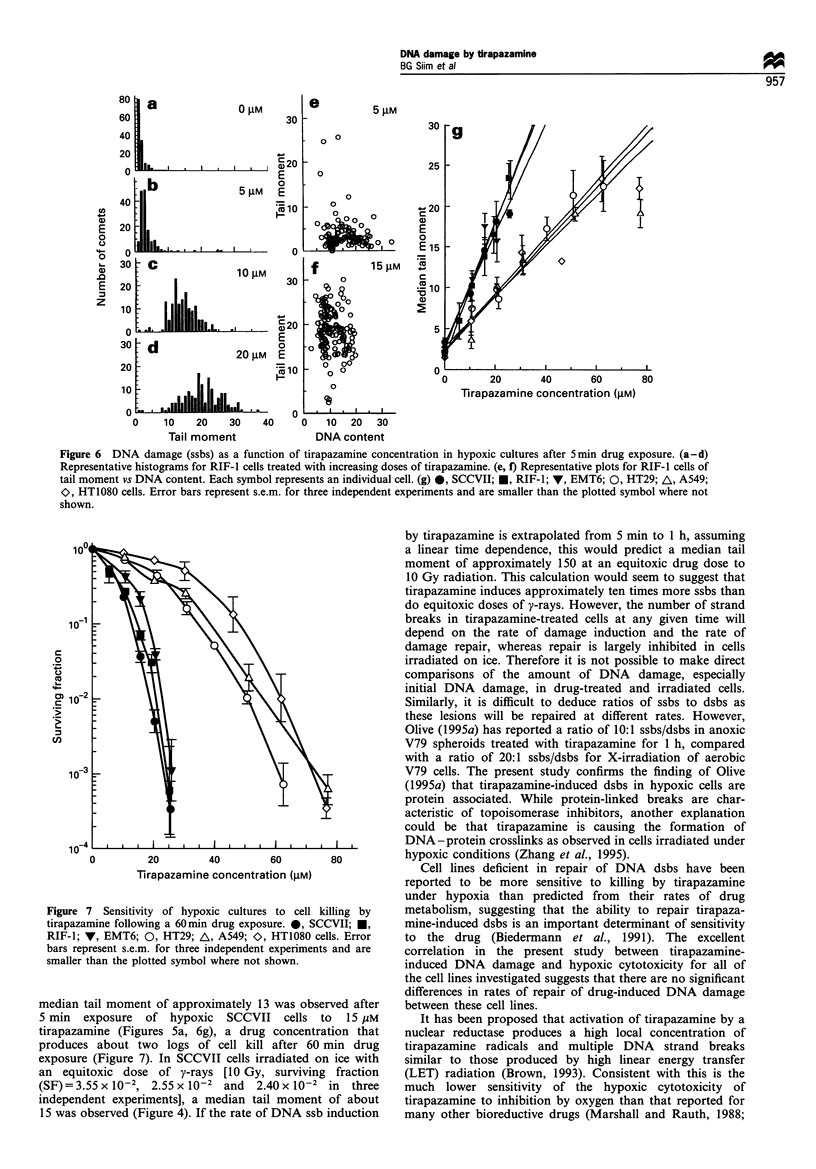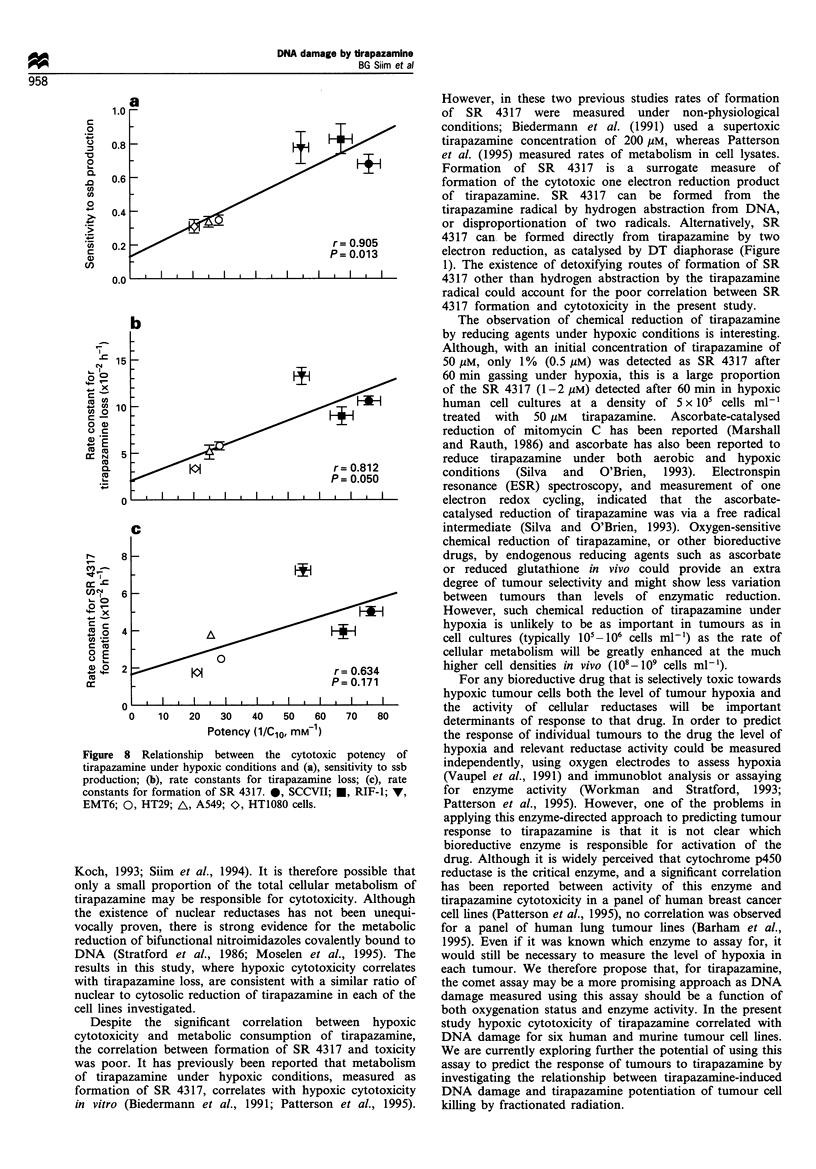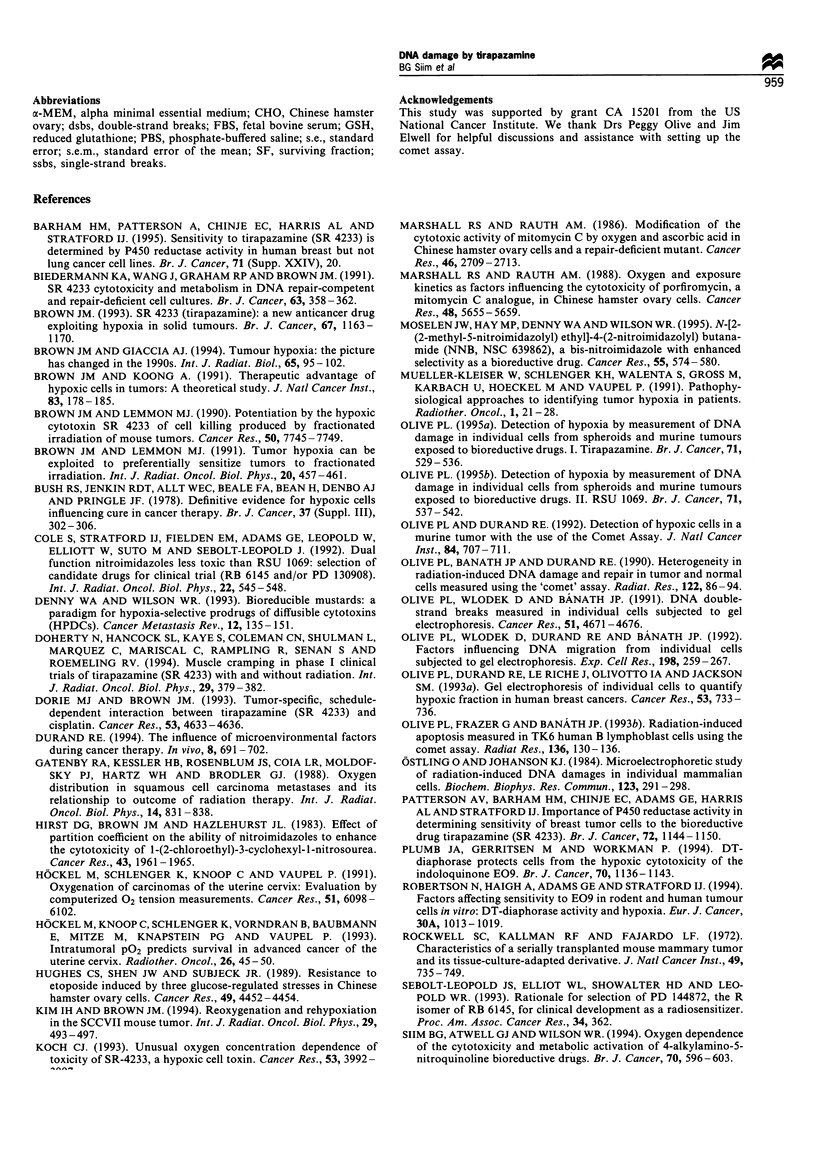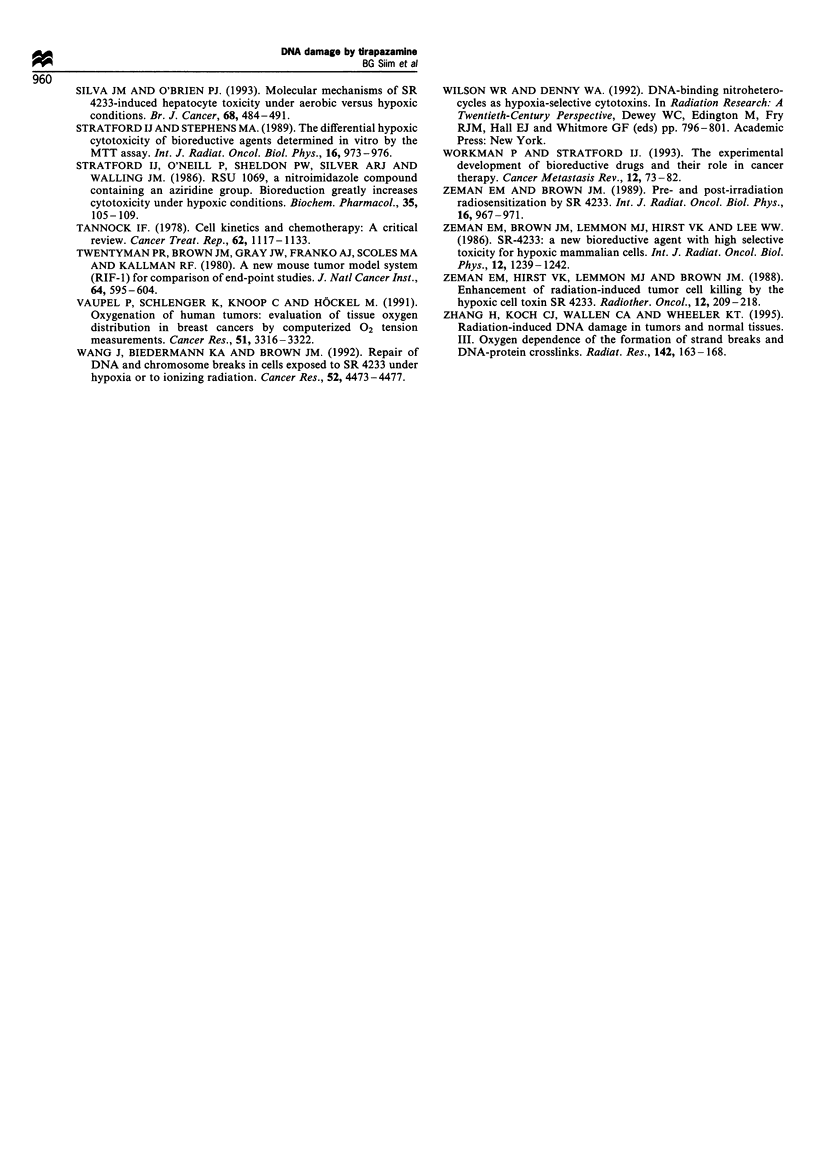# Tirapazamine-induced DNA damage measured using the comet assay correlates with cytotoxicity towards hypoxic tumour cells in vitro.

**DOI:** 10.1038/bjc.1996.187

**Published:** 1996-04

**Authors:** B. G. Siim, P. L. van Zijl, J. M. Brown

**Affiliations:** Department of Radiation Oncology, Stanford University School of Medicine, CA 94305-5468, USA.

## Abstract

Tirapazamine (SR 4233), a bioreductive drug selectively toxic towards hypoxic cells, is presently in phase II clinical trials. Since it would not be expected that all tumours would respond equally to the drug, we are exploring ways of predicting the response of individual tumours. In this study we have tested whether the comet assay, which measures DNA damage in individual cells, can provide a simple, surrogate end point for cell killing by tirapazamine. We examined the relationship between the cytotoxicity of tirapazamine under hypoxic conditions and tirapazamine-induced DNA strand breaks in murine (SCCVII, EMT6, RIF-1) and human (HT1080, A549, HT29) tumour cell lines. These results were compared with the relationship between tirapazamine cytotoxicity and another measure of the ability of cells to metabolise tirapazamine; high-performance liquid chromatography (HPLC) analysis of tirapazamine loss or formation of the two electron reduction product SR 4317. The correlation between the hypoxic cytotoxic potency of tirapazamine and DNA damage was highly significant (r = 0.905, P = 0.013). A similar correlation was observed for hypoxic potency and tirapazamine loss (r = 0.812, P = 0.050), while the correlation between hypoxic potency and SR 4317 formation was not significant (r = 0.634, P = 0.171). The hypoxic cytotoxicity of tirapazamine in vitro can therefore be predicted by measuring tirapazamine-induced DNA damage using the comet assay. This approach holds promise for predicting the response of individual tumours to tirapazamine in the clinic.


British Journal of Cancer (1996) 73, 952–960
© 1996 Stockton Press All rights reserved 0007–0920/96 $12.00

### Tirapazamine-induced DNA damage measured using the comet assay correlates with cytotoxicity towards hypoxic tumour cells *in vitro*

BG Siim, PL van Zijl and JM Brown

*Department of Radiation Oncology, Stanford University School of Medicine, Stanford, CA 94305–5468, USA.*

**Summary** Tirapazamine (SR 4233), a bioreductive drug selectively toxic towards hypoxic cells, is presently in phase II clinical trials. Since it would not be expected that all tumours would respond equally to the drug, we are exploring ways of predicting the response of individual tumours. In this study we have tested whether the comet assay, which measures DNA damage in individual cells, can provide a simple, surrogate end point for cell killing by tirapazamine. We examined the relationship between the cytotoxicity of tirapazamine under hypoxic conditions and tirapazamine-induced DNA strand breaks in murine (SCCVII, EMT6, RIF-1) and human (HT1080, A549, HT29) tumour cell lines. These results were compared with the relationship between tirapazamine cytotoxicity and another measure of the ability of cells to metabolise tirapazamine; high-performance liquid chromatography (HPLC) analysis of tirapazamine loss or formation of the two electron reduction product SR 4317. The correlation between the hypoxic cytotoxic potency of tirapazamine and DNA damage was highly significant ($r=0.905$, $P=0.013$). A similar correlation was observed for hypoxic potency and tirapazamine loss ($r=0.812$, $P=0.050$), while the correlation between hypoxic potency and SR 4317 formation was not significant ($r=0.634$, $P=0.171$). The hypoxic cytotoxicity of tirapazamine *in vitro* can therefore be predicted by measuring tirapazamine-induced DNA damage using the comet assay. This approach holds promise for predicting the response of individual tumours to tirapazamine in the clinic.

**Keywords:** tirapazamine; hypoxia; bioreductive drug; DNA damage; metabolism

Many human tumours contain a significant subpopulation of cells at low oxygen tensions (Höckel *et al.*, 1991; Mueller-Kleiser *et al.*, 1991; Vaupel *et al.*, 1991). These hypoxic cells, which can present a problem in the radiotherapy of solid tumours (Bush *et al.*, 1978; Gatenby *et al.*, 1988; Höckel *et al.*, 1993), are probably also resistant to many chemotherapeutic agents (Tannock, 1978; Hughes *et al.*, 1989; Wilson and Denny, 1992). It has recently been proposed that with the use of bioreductive drugs, which are selectively activated to a cytotoxic species under hypoxic conditions, it may be possible to exploit hypoxic tumour cells so that their presence in solid tumours is an advantage rather than a disadvantage (Brown and Koong, 1991; Denny and Wilson, 1993; Brown and Giaccia, 1994). This promising approach is about to be tested in the clinic with two new bioreductive drugs in, or about to enter, clinical trials; the benzotriazine di-*N*-oxide tirapazamine (SR 4233, WIN 59075) (Brown, 1993; Doherty *et al.*, 1994) and the dual-function alkylating nitroimidazole RB 6145 (or the less emetic R isomer PD 144872) (Cole *et al.*, 1992; Sebolt-Leopold *et al.*, 1993).

In the clinic it is expected that only patients with tumours containing a significant proportion of hypoxic cells would respond to a bioreductive drug that is selectively toxic towards hypoxic cells. The response of a tumour to any such bioreductive drug will depend both on the level of activity of the reductase(s) that activate the drug to a cytotoxic species, and the level of tumour hypoxia. Recent studies have shown that reductase activity can vary substantially between different human tumour cell lines (Plumb *et al.*, 1994; Robertson *et al.*, 1994), and measurements made using oxygen electrodes show large variations in oxygen tensions between tumours (Vaupel *et al.*, 1991). It would therefore be highly desirable to be able to predict the response of individual tumours to a bioreductive drug so that patients unlikely to benefit from the drug would not be treated with it, and further, the power of a clinical trial of the drug to produce a significant response would be improved.

Correspondence: JM Brown
Received 19 April 1995; revised 28 September 1995; accepted 20 November 1995

Tirapazamine exhibits high hypoxia-selective cytotoxicity in the order of 50 to 200-fold in most rodent and human cell lines (Zeman *et al.*, 1986, 1988; Stratford and Stephens, 1989). Tirapazamine also kills hypoxic cells in transplanted tumours in mice (Zeman *et al.*, 1988; Kim and Brown, 1994; Durand, 1994) and has been shown to potentiate the effects of modalities that are more toxic towards aerobic tumour cells. In particular, a large potentiation of tumour cell kill has been observed with fractionated radiation (Brown and Lemmon, 1990, 1991) and with some chemotherapeutic agents, particularly cisplatin (Dorie and Brown, 1993). Tirapazamine is currently in phase II clinical trials in combination with radiation and with cisplatin.

The metabolic activation of tirapazamine involves one electron reduction to a free radical species which, in the presence of oxygen, is back-oxidised to regenerate the parent drug with concomitant production of superoxide. Under hypoxic conditions the tirapazamine radical can abstract a hydrogen atom from DNA and produce a DNA strand break (Figure 1). The hypoxic cytotoxicity of tirapazamine is due to the formation of DNA double-strand breaks (dsbs) and resulting chromosome aberrations (Biedermann *et al.*, 1991; Wang *et al.*, 1992). Although it would be desirable to measure the cytotoxic lesion directly, it is probable that the low yield of dsbs in hypoxic cells treated with tirapazamine (Zeman and Brown, 1989; Olive, 1995*a*) would preclude measurement of dsbs using clinically achievable drug doses. We propose that the alkaline comet assay, which measures DNA single-strand breaks (ssbs) in individual cells (Olive *et al.*, 1990), may be used as a predictive assay for tumour response to tirapazamine. For this to hold, the ratio of DNA ssbs to dsbs would have to be constant across cell lines and the number of initial dsbs (and hence ssbs) predictive of cell kill. DNA damage measured using the comet assay should be a function of both the level of tumour hypoxia and reductase activity. In the present study we have assessed the relationship between tirapazamine-induced DNA damage and cytotoxicity in a number of murine (SCCVII, EMT6, RIF-1) and human (HT1080, A549, HT29) tumour cell lines treated with tirapazamine under hypoxic conditions. We have compared this with the correlation between hypoxic cytotoxicity and another measure of cells' ability to metabolise tirapazamine; high-performance liquid chromatography (HPLC) analysis of tirapazamine loss and formation of the two electron reduction product SR 4317 (Figure 1).

### Materials and methods

### *Cells and drugs*

Details of the derivation of the SCCVII (Hirst *et al.*, 1983), EMT6 (Rockwell *et al.*, 1972) and RIF-1 (Twentyman *et al.*, 1980) cell lines have been described previously. HT1080 and A549 cells were obtained from the American Type Culture Collection. HT29 cells were obtained from Dr RM Sutherland (SRI International, Menlo Park, CA, USA). Cells were cultured in Alpha MEM (HT1080, A549), McCoys 5A (HT29) or Waymouth's (SCCVII, EMT6, RIF-1) media supplemented with 10% (HT1080, A549, HT29) or 15% fetal bovine serum (FBS) (SCCVII, EMT6, RIF-1) plus penicillin (100 IU $ml^{-1}$) and streptomycin (100 $\mu g$ $ml^{-1}$). Tirapazamine was kindly supplied by Sterling Winthrop and SR 4317 and SR 4430 by SRI International.

### *Hypoxic drug exposures*

Hypoxic drug exposures were performed in double side-armed glass flasks (Wheaton Jacketed Reaction Vessels) at 37°C with continuous stirring using glass-coated magnetic stir bars. Tirapazamine solutions (9 ml), in alpha minimal essential medium (MEM) containing 10% FBS and antibiotics as above, at 1.25 times the final required drug concentration were equilibrated with humidified gas (nitrogen-5% carbon dioxide) for 60 min before sampling of 1 ml for HPLC analysis. Drug exposure was initiated by addition of cells (2 ml at $2.5 \times 10^6$ cells $ml^{-1}$ equilibrated under the same conditions). Samples of 0.5 ml ($2.5 \times 10^5$ cells) were taken after 5 min drug exposure and added to ice-cold phosphate-buffered saline (PBS) to give a final density of $2 \times 10^4$ cells $ml^{-1}$ for determination of DNA damage using the comet assay. After 60 min drug exposure, samples were taken to determine cytotoxicity and for HPLC analysis of tirapazamine and SR 4317 concentrations. Colony formation was assessed after incubation at 37°C for 8 (EMT6), 9 (SCCVII), 10 (HT1080), 12 (RIF-1, A549) or 14 days (HT29) by staining with 0.25% crystal violet in 95% ethanol and counting colonies containing >50 cells.

### *Comet assay*

The alkaline comet assay was performed as described previously by Olive and coworkers (Olive and Durand, 1992; Olive *et al.*, 1992). Briefly, 0.5 ml of cell suspension ($2 \times 10^4$ cells $ml^{-1}$ in cold PBS) was added to 1.5 ml of a 1% solution of low gelling temperature agarose (Sigma Type VII), mixed and pipetted onto a microscope slide on a cold block. The slides were placed in alkaline lysis solution (30 mM sodium hydroxide, 1 M sodium chloride, 0.1% *N*-lauroylsarcosine) for 60 min, then washed for $3 \times 20$ min in alkaline rinse solution (30 mM sodium hydroxide, 2 mM EDTA), followed by electrophoresis at 0.4 V $cm^{-1}$ for 25 min in a fresh solution of 30 mM sodium hydroxide, 2 mM EDTA. Slides were rinsed with distilled water for 15 min, then stained with propidium iodide (2.5 $\mu g$ $ml^{-1}$) for 15 min.

The neutral comet assay was performed as described by Olive (1995a). Slides prepared as above were placed in lysis solution (0.5% sodium dodecyl sulphate, 30 mM EDTA, pH 8.3) at 50°C for 4 h. Some slides were incubated overnight at 37°C with 0.5 mg $ml^{-1}$ proteinase K. After lysis slides were washed for at least 6 h in rinse buffer (90 mM tris, 90 mM boric acid, 2 mM EDTA, pH 8.5), followed by electrophoresis at 0.6 V $cm^{-1}$ for 25 min in fresh rinse buffer.

All slides were analysed within 48 h. The DNA from individual cells was visualised using a 20×objective with a Nikon Optiphot microscope attached to an Ikegami 4612 CCD camera and fluorescence image analysis system described by Olive *et al.* (1990). DNA damage was quantitated as the tail moment, the product of percentage of DNA in the tail and the mean tail length. A total of 150 comets were analysed per sample.

### *HPLC*

Metabolism of tirapazamine was assessed from HPLC analysis of samples of extracellular medium for parent drug and the two electron reduction product SR 4317. This provides an accurate assessment of cellular metabolism as the ratios of intracellular to extracellular concentrations for tirapazamine and SR 4317 are low (1.5–2 and 4–5 respectively in A549 cell cultures; PL van Zijl, unpublished data), and under the exposure conditions ($5 \times 10^5$ cells $ml^{-1}$) the extracellular volume is approximately $10^3$-fold greater than the intracellular volume, so the vast majority of the drug and reduction product will be in the extracellular compartment. The HPLC system consisted of a Waters pump (model 6000A), a WISP autosampler (model 712), a Waters Nova Pak-phenyl column (3.9×150 mm) and a Waters UV-visible detector (model 490E). The mobile phase was 22.5% methanol in water at a flow rate of 1.2 ml $min^{-1}$. Under these conditions tirapazamine, SR 4317 and SR 4330 had retention times of 3.7, 8.9 and 7.0 min respectively. Quantitation was based on peak areas using absorbance at 269 nm for tirapazamine and 238 nm for SR 4317 and SR 4330.

### Results

The cytotoxicity of tirapazamine towards three human (HT1080, A549, HT29) and three murine (SCCVII, RIF-1, EMT6) tumour cell lines was assessed under hypoxic conditions and correlated with three measures of drug metabolism determined in the same experiments: production of tirapazamine-induced DNA strand breaks, loss of tirapazamine and formation of SR 4317.

### *HPLC analysis of metabolism*

Tirapazamine solutions were equilibrated with nitrogen-5% carbon dioxide for 60 min before initiation of drug exposure by addition of cells equilibrated under identical conditions. Before mixing, samples were taken for HPLC analysis to check for evaporative loss of water from the drug solutions. There was consistent, reproducible metabolism of tirapazamine in media gassed under hypoxic conditions, as indicated by detection of the two-electron reduction product SR 4317. For an initial drug concentration of 50 $\mu$M, approximately 0.5 $\mu$M SR 4317 was detected following 60 min gassing under hypoxic conditions, with a 2-fold higher concentration detected after 120 min (Table I). This metabolism in the absence of cells was via the oxygen-sensitive one-electron reduction product as there was complete inhibition of

**Table I** Chemical reduction of tirapazamine (50 $\mu$M) under hypoxic conditions

| | SR 4317 ($\mu$M)[a] | |
|---|---|---|
| *Solvent* | *60 min* | *120 min* |
| Alpha MEM + 10% FBS | 0.45 ± 0.04[b] | 1.00 ± 0.07[b] |
| Alpha MEM no FBS | 0.44 ± 0.13[b] | 1.05 ± 0.41[b] |
| PBS | 0.02 ± 0.01 | 0.03 ± 0.01 |
| PBS + 10% FBS | 0.13 ± 0.01 | 0.21 ± 0.02 |
| PBS + 50 mg $l^{-1}$ ascorbate | 0.24 ± 0.02 | 0.46 ± 0.06 |
| PBS + 100 mg $l^{-1}$ cysteine | 0.71 ± 0.23 | 0.90 ± 0.25 |
| PBS + 10% FBS + 50 mg $l^{-1}$ ascorbate + 100 mg $l^{-1}$ cysteine | 0.24 ± 0.08 | 0.51 ± 0.19 |
| PBS + 5 mM GSH | 0.47 ± 0.13 | 0.70 ± 0.16 |

[a]Values are means ± s.e.m. for three independent experiments.
[b]Values are ranges for two independent experiments.

metabolism when tirapazamine was gassed under aerobic conditions. Metabolism was also inhibited in PBS, although addition of FBS, cysteine or ascorbate to PBS at the concentrations present in α-MEM each resulted in some metabolism of tirapazamine, which increased with time of hypoxic gassing (Table I). It thus appears that there are a number of reducing agents present in α-MEM that can chemically reduce tirapazamine under hypoxic conditions. Rates of cellular metabolism of tirapazamine were therefore corrected for this chemical reduction. Reduced glutathione, a reducing agent present in high concentrations in most cells, can also reduce tirapazamine under hypoxic conditions (Table I).

The rate of metabolism of tirapazamine in hypoxic cultures, as measured by HPLC analysis of tirapazamine loss, or formation of SR 4317 in the extracellular media, increased linearly with tirapazamine concentration in each of the cell lines investigated (Figure 2a,b). The rate of tirapazamine loss decreased in the order EMT6 > SCCVII > RIF-1 > HT29 > A549 > HT1080 (Figure 2a). Rates of formation of SR 4317 followed a similar pattern; EMT6 > SCCVII > RIF-1 ≈ A549 > HT29 > HT1080 (Figure 2b). The amount of SR 4317 detected in hypoxic A549 cultures was high relative to the amount of tirapazamine metabolised. In A549 cells about 70% of the tirapazamine metabolised was detected as SR 4317, compared with about 45% for the other cell lines. No SR 4330, the four electron reduction product of tirapazamine, was detected in any of the samples.

*DNA damage (comet assay)*

The neutral comet assay was used to measure DNA dsbs in hypoxic SCCVII cells treated with tirapazamine for 60 min. For a tirapazamine concentration of 20 μM, which produces approximately 2.5 logs of cell killing after 60 min drug exposure, there was essentially no increase in DNA damage above control levels either with or without proteinase K (Figure 3). In the absence of proteinase K no damage was detected even at drug concentrations of up to 200 μM. In contrast, incubation with proteinase K provided a linear increase in DNA damage with tirapazamine concentration.

Initial experiments with the alkaline comet assay confirmed that there was a linear relationship between DNA damage (strand breaks) and radiation dose up to 20 Gy for SCCVII and A549 cells (Figure 4). In hypoxic SCCVII cells treated with 15 μM tirapazamine (a drug concentration that gives approximately 2 logs of cell kill after 60 min exposure) DNA damage increased linearly with time of drug exposure at early times before appearing to saturate after about 10 min exposure (Figure 5a). The apparent saturation of damage appears to reflect the upper sensitivity limit for the comet assay under the conditions described earlier. Comets from highly damaged cells have very little DNA in the head of the comet and consequently are not recognised by the image analysis program. Tirapazamine-induced DNA damage was therefore assessed in all further experiments after 5 min drug exposure. However, recent experiments with hypoxic SCCVII cells and lower tirapazamine concentrations have indicated that DNA damage reaches an equilibrium after 10 min drug exposure that is dependent on the drug concentration (data not shown). The basis for this is currently under investigation. In hypoxic SCCVII cells DNA damage measured either 5 or 60 min after initiation of drug exposure showed a similar relationship to cytotoxicity determined after 60 min (Figure 5b).

As DNA damage was not normally distributed (Figure 6a–d), we have used the median tail moment as being representative of average damage. Tail moment was independent of DNA content, calculated from total cellular fluorescence (Figure 6e and f), indicating there is no cell cycle specificity of DNA damage. In each of the cell lines investigated DNA damage induced by tirapazamine under hypoxic conditions increased linearly with drug concentration (Figure 6g), with the exception of the highest concentrations in the human cell lines where damage appeared to saturate, although cytotoxicity still increased (Figure 7). The murine cell lines all had similar sensitivities to induction of ssbs by tirapazamine, as did the human cell lines. For a given tirapazamine concentration there was about 2-fold greater DNA damage in the murine than in the human lines (Figure 6g).

*Cytotoxicity*

The cytotoxicity of tirapazamine towards hypoxic cell cultures following a 60 min drug exposure was assessed by

**Figure 1** Scheme showing metabolic activation of tirapazamine. One electron reduction gives a radical species that is back-oxidised in the presence of oxygen. Under hypoxic conditions the radical can abstract a hydrogen atom from DNA to produce a strand break and SR 4317. SR 4317 can also be formed by disproportionation of two tirapazamine radicals or directly from tirapazamine by two electron reduction. Hypoxic cell death results from formation of DNA dsbs. This study compares the correlation between DNA strand break production and hypoxic cytotoxicity with the correlation between loss of tirapazamine or formation of SR 4317 and cytotoxicity.

clonogenic survival curves over a range of drug concentrations (Figure 7). The murine tumour cell lines all had similar sensitivities to cell killing by tirapazamine under hypoxic conditions, and were 2- to 3-fold more sensitive than the human cell lines.

*Relationship between sensitivity to tirapazamine and DNA damage*

As DNA damage was linearly related to tirapazamine concentration (Figure 6g), the sensitivity to ssb production was measured from the slope of the dose–response curves. These values were plotted against hypoxic cytotoxic potency, calculated as the reciprocal of the $C_{10}$ value (drug concentration required to reduce survival to 10%) obtained by interpolation from Figure 7. There was a highly significant correlation ($r = 0.905$, $P = 0.013$) between sensitivity to ssb production and hypoxic potency (Figure 8a). The potency of tirapazamine towards the human cell lines was 2- to 3-fold lower than towards the murine lines, with tirapazamine producing 2- to 3-fold less DNA damage in the human than in the murine lines.

**Figure 2** Rate of metabolism of tirapazamine in hypoxic cultures as a function of initial drug concentration. (**a**) Tirapazamine loss. (**b**) Formation of SR 4317. ●, SCCVII; ■, RIF-1; ▼, EMT6; ○, HT29; △, A549; ◇, HT1080 cells. Error bars represent s.e.m. for three independent experiments and are smaller than the plotted symbol where not shown.

**Figure 3** DNA damage (dsbs) measured using the neutral comet assay after 60 min exposure of hypoxic SCCVII cells to tirapazamine. (○) No proteinase K; (●) overnight incubation with proteinase K. The mean ± s.e. for 150 comets is shown for each dose.

**Figure 4** DNA damage measured using the alkaline comet assay as a function of radiation dose in SCCVII (●) and A549 (△) cells irradiated on ice. The mean ± s.e. for 150 comets is shown for each dose.

*Relationship between sensitivity to tirapazamine and metabolism*

First-order rate constants for loss of tirapazamine or formation of SR 4317 were calculated from the slopes of the plots in Figure 2 and plotted against hypoxic cytotoxic potency (Figures 8b and c). There was a significant correlation ($r=0.812$, $P=0.050$) between the rate constants for tirapazamine loss and hypoxic potency, with tirapazamine being most potent against the SCCVII, RIF-1 and EMT6 cell lines and being metabolised fastest in those lines (Figure 8b).

**Figure 5** **(a)** Time course for formation of DNA ssbs in hypoxic SCCVII cells treated with 15 $\mu$M tirapazamine. **(b)** Relationship between DNA damage (tail moment) measured after 5 (■) or 60 (○) min exposure of hypoxic SCCVII cells to tirapazamine, and cell survival after 60 min. The mean ± s.e. for 150 comets is shown for each point.

While the rate constants for SR 4317 formation increased with potency (Figure 8c), the correlation was not significant ($r=0.634$, $P=0.171$).

### Discussion

This study is the first step in determining whether the comet assay can be used to predict tumour response to tirapazamine. Olive and colleagues have adapted the single-cell gel electrophoresis method described by Östling and Johanson (1984) to video image analysis and reported many applications for the comet assay, including the detection of radiation-induced apoptosis (Olive *et al.*, 1993*b*), DNA dsbs (Olive *et al.*, 1991), and hypoxia in murine tumours (Olive and Durand, 1992; Olive, 1995*a*,*b*) and in human breast tumours (Olive *et al.*, 1993*a*). The mechanism of hypoxic cytotoxicity of tirapazamine, by the formation of DNA dsbs and resulting chromosome breaks, suggested to us that the comet assay, which measures DNA strand breaks in individual cells, may predict for cell killing by tirapazamine under hypoxic conditions. While it would be preferable to measure the cytotoxic lesion (DNA dsbs) directly, the low yield of such lesions necessitates the use of supertoxic drug doses (Figure 3), which would clearly preclude the use of such an assay in clinical situations. The present study demonstrates a good correlation between tirapazamine-induced DNA ssbs and hypoxic cell killing by tirapazamine *in vitro*, suggesting fulfillment of the requirement that the ratio of DNA ssbs to dsbs would have to be constant across cell lines, and the number of initial dsbs and ssbs predictive of cell kill.

The observed 2 to 3-fold greater sensitivity of the murine tumour cell lines to killing by tirapazamine under hypoxic conditions compared with the human lines is consistent with previously published data (Zeman *et al.*, 1986; Biedermann *et al.*, 1991) and appears to underlie the lower hypoxic cytotoxicity ratios (ratio of drug concentrations required to give the same level of killing under aerobic and hypoxic conditions) reported for tirapazamine in human than in murine cell lines (Zeman *et al.*, 1986). It should be recognised that, although the hypoxic selective cytotoxicity of tirapazamine is generally lower in human than murine cell lines, it is still substantial, with hypoxic cytotoxicity ratios of around 50 being common (Zeman *et al.*, 1986). Tirapazamine induced 2- to 3-fold more DNA damage in the hypoxic murine cell lines than in the human cell lines, indicating that the variation in hypoxic sensitivity could be entirely accounted for by the production of DNA strand breaks.

From the limited number of cell lines in the present study it appears that the correlation between DNA damage and cell survival could largely result from interspecies differences. The similar DNA damage and sensitivity of each of the human and each of the murine cell lines suggests that they must possess similar levels of activity of the reductase(s) that activates tirapazamine to a cytotoxic species. However, in the clinic, wide variations in reductase activity, as has been reported for human tumour cell lines (Plumb *et al.*, 1994; Robertson *et al.*, 1994), and varying levels of hypoxia (Vaupel *et al.*, 1991), would be expected to result in large differences in sensitivity to tirapazamine. It is of primary importance that in this study, by measuring DNA damage, it was possible to distinguish between cell lines with 2- to 3-fold differences in sensitivity to killing by tirapazamine under hypoxic conditions. Although we measured initial DNA damage after 5 min drug exposure, when the effects of repair should be minimal, and showed this to correlate with cytotoxicity after 60 min, it appears that, at least for the SCCVII cell line, DNA damage after 60 min also correlates with cytotoxicity (Figure 5b). This augurs well for when this approach is tested *in vivo* in animal models, or in the clinic, as in these situations it will not be possible to measure initial DNA damage.

In the present study high numbers of ssbs were produced very rapidly in hypoxic cells treated with tirapazamine. A

**Figure 6** DNA damage (ssbs) as a function of tirapazamine concentration in hypoxic cultures after 5 min drug exposure. (**a**–**d**) Representative histograms for RIF-1 cells treated with increasing doses of tirapazamine. (**e**, **f**) Representative plots for RIF-1 cells of tail moment *vs* DNA content. Each symbol represents an individual cell. (**g**) ●, SCCVII; ■, RIF-1; ▼, EMT6; ○, HT29; △, A549; ◇, HT1080 cells. Error bars represent s.e.m. for three independent experiments and are smaller than the plotted symbol where not shown.

**Figure 7** Sensitivity of hypoxic cultures to cell killing by tirapazamine following a 60 min drug exposure. ●, SCCVII; ■, RIF-1; ▼, EMT6; ○, HT29; △, A549; ◇, HT1080 cells. Error bars represent s.e.m. for three independent experiments and are smaller than the plotted symbol where not shown.

median tail moment of approximately 13 was observed after 5 min exposure of hypoxic SCCVII cells to 15 μM tirapazamine (Figures 5a, 6g), a drug concentration that produces about two logs of cell kill after 60 min drug exposure (Figure 7). In SCCVII cells irradiated on ice with an equitoxic dose of γ-rays [10 Gy, surviving fraction (SF) = $3.55 \times 10^{-2}$, $2.55 \times 10^{-2}$ and $2.40 \times 10^{-2}$ in three independent experiments], a median tail moment of about 15 was observed (Figure 4). If the rate of DNA ssb induction by tirapazamine is extrapolated from 5 min to 1 h, assuming a linear time dependence, this would predict a median tail moment of approximately 150 at an equitoxic drug dose to 10 Gy radiation. This calculation would seem to suggest that tirapazamine induces approximately ten times more ssbs than do equitoxic doses of γ-rays. However, the number of strand breaks in tirapazamine-treated cells at any given time will depend on the rate of damage induction and the rate of damage repair, whereas repair is largely inhibited in cells irradiated on ice. Therefore it is not possible to make direct comparisons of the amount of DNA damage, especially initial DNA damage, in drug-treated and irradiated cells. Similarly, it is difficult to deduce ratios of ssbs to dsbs as these lesions will be repaired at different rates. However, Olive (1995*a*) has reported a ratio of 10:1 ssbs/dsbs in anoxic V79 spheroids treated with tirapazamine for 1 h, compared with a ratio of 20:1 ssbs/dsbs for X-irradiation of aerobic V79 cells. The present study confirms the finding of Olive (1995*a*) that tirapazamine-induced dsbs in hypoxic cells are protein associated. While protein-linked breaks are characteristic of topoisomerase inhibitors, another explanation could be that tirapazamine is causing the formation of DNA–protein crosslinks as observed in cells irradiated under hypoxic conditions (Zhang *et al.*, 1995).

Cell lines deficient in repair of DNA dsbs have been reported to be more sensitive to killing by tirapazamine under hypoxia than predicted from their rates of drug metabolism, suggesting that the ability to repair tirapazamine-induced dsbs is an important determinant of sensitivity to the drug (Biedermann *et al.*, 1991). The excellent correlation in the present study between tirapazamine-induced DNA damage and hypoxic cytotoxicity for all of the cell lines investigated suggests that there are no significant differences in rates of repair of drug-induced DNA damage between these cell lines.

It has been proposed that activation of tirapazamine by a nuclear reductase produces a high local concentration of tirapazamine radicals and multiple DNA strand breaks similar to those produced by high linear energy transfer (LET) radiation (Brown, 1993). Consistent with this is the much lower sensitivity of the hypoxic cytotoxicity of tirapazamine to inhibition by oxygen than that reported for many other bioreductive drugs (Marshall and Rauth, 1988;

**Figure 8** Relationship between the cytotoxic potency of tirapazamine under hypoxic conditions and (**a**), sensitivity to ssb production; (**b**), rate constants for tirapazamine loss; (**c**), rate constants for formation of SR 4317. ●, SCCVII; ■, RIF-1; ▼, EMT6; ○, HT29; △, A549; ◇, HT1080 cells.

Koch, 1993; Siim *et al.*, 1994). It is therefore possible that only a small proportion of the total cellular metabolism of tirapazamine may be responsible for cytotoxicity. Although the existence of nuclear reductases has not been unequivocally proven, there is strong evidence for the metabolic reduction of bifunctional nitroimidazoles covalently bound to DNA (Stratford *et al.*, 1986; Moselen *et al.*, 1995). The results in this study, where hypoxic cytotoxicity correlates with tirapazamine loss, are consistent with a similar ratio of nuclear to cytosolic reduction of tirapazamine in each of the cell lines investigated.

Despite the significant correlation between hypoxic cytotoxicity and metabolic consumption of tirapazamine, the correlation between formation of SR 4317 and toxicity was poor. It has previously been reported that metabolism of tirapazamine under hypoxic conditions, measured as formation of SR 4317, correlates with hypoxic cytotoxicity *in vitro* (Biedermann *et al.*, 1991; Patterson *et al.*, 1995). However, in these two previous studies rates of formation of SR 4317 were measured under non-physiological conditions; Biedermann *et al.* (1991) used a supertoxic tirapazamine concentration of 200 μM, whereas Patterson *et al.* (1995) measured rates of metabolism in cell lysates. Formation of SR 4317 is a surrogate measure of formation of the cytotoxic one electron reduction product of tirapazamine. SR 4317 can be formed from the tirapazamine radical by hydrogen abstraction from DNA, or disproportionation of two radicals. Alternatively, SR 4317 can be formed directly from tirapazamine by two electron reduction, as catalysed by DT diaphorase (Figure 1). The existence of detoxifying routes of formation of SR 4317 other than hydrogen abstraction by the tirapazamine radical could account for the poor correlation between SR 4317 formation and cytotoxicity in the present study.

The observation of chemical reduction of tirapazamine by reducing agents under hypoxic conditions is interesting. Although, with an initial concentration of tirapazamine of 50 μM, only 1% (0.5 μM) was detected as SR 4317 after 60 min gassing under hypoxia, this is a large proportion of the SR 4317 (1–2 μM) detected after 60 min in hypoxic human cell cultures at a density of $5 \times 10^5$ cells ml$^{-1}$ treated with 50 μM tirapazamine. Ascorbate-catalysed reduction of mitomycin C has been reported (Marshall and Rauth, 1986) and ascorbate has also been reported to reduce tirapazamine under both aerobic and hypoxic conditions (Silva and O'Brien, 1993). Electronspin resonance (ESR) spectroscopy, and measurement of one electron redox cycling, indicated that the ascorbate-catalysed reduction of tirapazamine was via a free radical intermediate (Silva and O'Brien, 1993). Oxygen-sensitive chemical reduction of tirapazamine, or other bioreductive drugs, by endogenous reducing agents such as ascorbate or reduced glutathione *in vivo* could provide an extra degree of tumour selectivity and might show less variation between tumours than levels of enzymatic reduction. However, such chemical reduction of tirapazamine under hypoxia is unlikely to be as important in tumours as in cell cultures (typically $10^5-10^6$ cells ml$^{-1}$) as the rate of cellular metabolism will be greatly enhanced at the much higher cell densities *in vivo* ($10^8-10^9$ cells ml$^{-1}$).

For any bioreductive drug that is selectively toxic towards hypoxic tumour cells both the level of tumour hypoxia and the activity of cellular reductases will be important determinants of response to that drug. In order to predict the response of individual tumours to the drug the level of hypoxia and relevant reductase activity could be measured independently, using oxygen electrodes to assess hypoxia (Vaupel *et al.*, 1991) and immunoblot analysis or assaying for enzyme activity (Workman and Stratford, 1993; Patterson *et al.*, 1995). However, one of the problems in applying this enzyme-directed approach to predicting tumour response to tirapazamine is that it is not clear which bioreductive enzyme is responsible for activation of the drug. Although it is widely perceived that cytochrome p450 reductase is the critical enzyme, and a significant correlation has been reported between activity of this enzyme and tirapazamine cytotoxicity in a panel of human breast cancer cell lines (Patterson *et al.*, 1995), no correlation was observed for a panel of human lung tumour lines (Barham *et al.*, 1995). Even if it was known which enzyme to assay for, it would still be necessary to measure the level of hypoxia in each tumour. We therefore propose that, for tirapazamine, the comet assay may be a more promising approach as DNA damage measured using this assay should be a function of both oxygenation status and enzyme activity. In the present study hypoxic cytotoxicity of tirapazamine correlated with DNA damage for six human and murine tumour cell lines. We are currently exploring further the potential of using this assay to predict the response of tumours to tirapazamine by investigating the relationship between tirapazamine-induced DNA damage and tirapazamine potentiation of tumour cell killing by fractionated radiation.

**Abbreviations**

α-MEM, alpha minimal essential medium; CHO, Chinese hamster ovary; dsbs, double-strand breaks; FBS, fetal bovine serum; GSH, reduced glutathione; PBS, phosphate-buffered saline; s.e., standard error; s.e.m., standard error of the mean; SF, surviving fraction; ssbs, single-strand breaks.

**Acknowledgements**

This study was supported by grant CA 15201 from the US National Cancer Institute. We thank Drs Peggy Olive and Jim Elwell for helpful discussions and assistance with setting up the comet assay.

### References

BARHAM HM, PATTERSON A, CHINJE EC, HARRIS AL AND STRATFORD IJ. (1995). Sensitivity to tirapazamine (SR 4233) is determined by P450 reductase activity in human breast but not lung cancer cell lines. *Br. J. Cancer*, **71** (Supp. XXIV), 20.

BIEDERMANN KA, WANG J, GRAHAM RP AND BROWN JM. (1991). SR 4233 cytotoxicity and metabolism in DNA repair-competent and repair-deficient cell cultures. *Br. J. Cancer*, **63**, 358–362.

BROWN JM. (1993). SR 4233 (tirapazamine): a new anticancer drug exploiting hypoxia in solid tumours. *Br. J. Cancer*, **67**, 1163–1170.

BROWN JM AND GIACCIA AJ. (1994). Tumour hypoxia: the picture has changed in the 1990s. *Int. J. Radiat. Biol.*, **65**, 95–102.

BROWN JM AND KOONG A. (1991). Therapeutic advantage of hypoxic cells in tumors: A theoretical study. *J. Natl Cancer Inst.*, **83**, 178–185.

BROWN JM AND LEMMON MJ. (1990). Potentiation by the hypoxic cytotoxin SR 4233 of cell killing produced by fractionated irradiation of mouse tumors. *Cancer Res.*, **50**, 7745–7749.

BROWN JM AND LEMMON MJ. (1991). Tumor hypoxia can be exploited to preferentially sensitize tumors to fractionated irradiation. *Int. J. Radiat. Oncol. Biol. Phys.*, **20**, 457–461.

BUSH RS, JENKIN RDT, ALLT WEC, BEALE FA, BEAN H, DENBO AJ AND PRINGLE JF. (1978). Definitive evidence for hypoxic cells influencing cure in cancer therapy. *Br. J. Cancer*, **37** (Suppl. III), 302–306.

COLE S, STRATFORD IJ, FIELDEN EM, ADAMS GE, LEOPOLD W, ELLIOTT W, SUTO M AND SEBOLT-LEOPOLD J. (1992). Dual function nitroimidazoles less toxic than RSU 1069: selection of candidate drugs for clinical trial (RB 6145 and/or PD 130908). *Int. J. Radiat. Oncol. Biol. Phys.*, **22**, 545–548.

DENNY WA AND WILSON WR. (1993). Bioreducible mustards: a paradigm for hypoxia-selective prodrugs of diffusible cytotoxins (HPDCs). *Cancer Metastasis Rev.*, **12**, 135–151.

DOHERTY N, HANCOCK SL, KAYE S, COLEMAN CN, SHULMAN L, MARQUEZ C, MARISCAL C, RAMPLING R, SENAN S AND ROEMELING RV. (1994). Muscle cramping in phase I clinical trials of tirapazamine (SR 4233) with and without radiation. *Int. J. Radiat. Oncol. Biol. Phys.*, **29**, 379–382.

DORIE MJ AND BROWN JM. (1993). Tumor-specific, schedule-dependent interaction between tirapazamine (SR 4233) and cisplatin. *Cancer Res.*, **53**, 4633–4636.

DURAND RE. (1994). The influence of microenvironmental factors during cancer therapy. *In vivo*, **8**, 691–702.

GATENBY RA, KESSLER HB, ROSENBLUM JS, COIA LR, MOLDOFSKY PJ, HARTZ WH AND BRODLER GJ. (1988). Oxygen distribution in squamous cell carcinoma metastases and its relationship to outcome of radiation therapy. *Int. J. Radiat. Oncol. Biol. Phys.*, **14**, 831–838.

HIRST DG, BROWN JM AND HAZLEHURST JL. (1983). Effect of partition coefficient on the ability of nitroimidazoles to enhance the cytotoxicity of 1-(2-chloroethyl)-3-cyclohexyl-1-nitrosourea. *Cancer Res.*, **43**, 1961–1965.

HÖCKEL M, SCHLENGER K, KNOOP C AND VAUPEL P. (1991). Oxygenation of carcinomas of the uterine cervix: Evaluation by computerized $O_2$ tension measurements. *Cancer Res.*, **51**, 6098–6102.

HÖCKEL M, KNOOP C, SCHLENGER K, VORNDRAN B, BAUBMANN E, MITZE M, KNAPSTEIN PG AND VAUPEL P. (1993). Intratumoral $pO_2$ predicts survival in advanced cancer of the uterine cervix. *Radiother. Oncol.*, **26**, 45–50.

HUGHES CS, SHEN JW AND SUBJECK JR. (1989). Resistance to etoposide induced by three glucose-regulated stresses in Chinese hamster ovary cells. *Cancer Res.*, **49**, 4452–4454.

KIM IH AND BROWN JM. (1994). Reoxygenation and rehypoxiation in the SCCVII mouse tumor. *Int. J. Radiat. Oncol. Biol. Phys.*, **29**, 493–497.

KOCH CJ. (1993). Unusual oxygen concentration dependence of toxicity of SR-4233, a hypoxic cell toxin. *Cancer Res.*, **53**, 3992–[illegible]

MARSHALL RS AND RAUTH AM. (1986). Modification of the cytotoxic activity of mitomycin C by oxygen and ascorbic acid in Chinese hamster ovary cells and a repair-deficient mutant. *Cancer Res.*, **46**, 2709–2713.

MARSHALL RS AND RAUTH AM. (1988). Oxygen and exposure kinetics as factors influencing the cytotoxicity of porfiromycin, a mitomycin C analogue, in Chinese hamster ovary cells. *Cancer Res.*, **48**, 5655–5659.

MOSELEN JW, HAY MP, DENNY WA AND WILSON WR. (1995). *N*-[2-(2-methyl-5-nitroimidazolyl) ethyl]-4-(2-nitroimidazolyl) butanamide (NNB, NSC 639862), a bis-nitroimidazole with enhanced selectivity as a bioreductive drug. *Cancer Res.*, **55**, 574–580.

MUELLER-KLEISER W, SCHLENGER KH, WALENTA S, GROSS M, KARBACH U, HOECKEL M AND VAUPEL P. (1991). Pathophysiological approaches to identifying tumor hypoxia in patients. *Radiother. Oncol.*, **1**, 21–28.

OLIVE PL. (1995*a*). Detection of hypoxia by measurement of DNA damage in individual cells from spheroids and murine tumours exposed to bioreductive drugs. I. Tirapazamine. *Br. J. Cancer*, **71**, 529–536.

OLIVE PL. (1995*b*). Detection of hypoxia by measurement of DNA damage in individual cells from spheroids and murine tumours exposed to bioreductive drugs. II. RSU 1069. *Br. J. Cancer*, **71**, 537–542.

OLIVE PL AND DURAND RE. (1992). Detection of hypoxic cells in a murine tumor with the use of the Comet Assay. *J. Natl Cancer Inst.*, **84**, 707–711.

OLIVE PL, BANATH JP AND DURAND RE. (1990). Heterogeneity in radiation-induced DNA damage and repair in tumor and normal cells measured using the 'comet' assay. *Radiat. Res.*, **122**, 86–94.

OLIVE PL, WLODEK D AND BÁNATH JP. (1991). DNA double-strand breaks measured in individual cells subjected to gel electrophoresis. *Cancer Res.*, **51**, 4671–4676.

OLIVE PL, WLODEK D, DURAND RE AND BÁNATH JP. (1992). Factors influencing DNA migration from individual cells subjected to gel electrophoresis. *Exp. Cell Res.*, **198**, 259–267.

OLIVE PL, DURAND RE, LE RICHE J, OLIVOTTO IA AND JACKSON SM. (1993*a*). Gel electrophoresis of individual cells to quantify hypoxic fraction in human breast cancers. *Cancer Res.*, **53**, 733–736.

OLIVE PL, FRAZER G AND BANÁTH JP. (1993*b*). Radiation-induced apoptosis measured in TK6 human B lymphoblast cells using the comet assay. *Radiat Res.*, **136**, 130–136.

ÖSTLING O AND JOHANSON KJ. (1984). Microelectrophoretic study of radiation-induced DNA damages in individual mammalian cells. *Biochem. Biophys. Res. Commun.*, **123**, 291–298.

PATTERSON AV, BARHAM HM, CHINJE EC, ADAMS GE, HARRIS AL AND STRATFORD IJ. Importance of P450 reductase activity in determining sensitivity of breast tumor cells to the bioreductive drug tirapazamine (SR 4233). *Br. J. Cancer*, **72**, 1144–1150.

PLUMB JA, GERRITSEN M AND WORKMAN P. (1994). DT-diaphorase protects cells from the hypoxic cytotoxicity of the indoloquinone EO9. *Br. J. Cancer*, **70**, 1136–1143.

ROBERTSON N, HAIGH A, ADAMS GE AND STRATFORD IJ. (1994). Factors affecting sensitivity to EO9 in rodent and human tumour cells *in vitro*: DT-diaphorase activity and hypoxia. *Eur. J. Cancer*, **30A**, 1013–1019.

ROCKWELL SC, KALLMAN RF AND FAJARDO LF. (1972). Characteristics of a serially transplanted mouse mammary tumor and its tissue-culture-adapted derivative. *J. Natl Cancer Inst.*, **49**, 735–749.

SEBOLT-LEOPOLD JS, ELLIOT WL, SHOWALTER HD AND LEOPOLD WR. (1993). Rationale for selection of PD 144872, the R isomer of RB 6145, for clinical development as a radiosensitizer. *Proc. Am. Assoc. Cancer Res.*, **34**, 362.

SIIM BG, ATWELL GJ AND WILSON WR. (1994). Oxygen dependence of the cytotoxicity and metabolic activation of 4-alkylamino-5-nitroquinoline bioreductive drugs. *Br. J. Cancer*, **70**, 596–603.

SILVA JM AND O'BRIEN PJ. (1993). Molecular mechanisms of SR 4233-induced hepatocyte toxicity under aerobic versus hypoxic conditions. *Br. J. Cancer*, **68**, 484–491.

STRATFORD IJ AND STEPHENS MA. (1989). The differential hypoxic cytotoxicity of bioreductive agents determined in vitro by the MTT assay. *Int. J. Radiat. Oncol. Biol. Phys.*, **16**, 973–976.

STRATFORD IJ, O'NEILL P, SHELDON PW, SILVER ARJ AND WALLING JM. (1986). RSU 1069, a nitroimidazole compound containing an aziridine group. Bioreduction greatly increases cytotoxicity under hypoxic conditions. *Biochem. Pharmacol.*, **35**, 105–109.

TANNOCK IF. (1978). Cell kinetics and chemotherapy: A critical review. *Cancer Treat. Rep.*, **62**, 1117–1133.

TWENTYMAN PR, BROWN JM, GRAY JW, FRANKO AJ, SCOLES MA AND KALLMAN RF. (1980). A new mouse tumor model system (RIF-1) for comparison of end-point studies. *J. Natl Cancer Inst.*, **64**, 595–604.

VAUPEL P, SCHLENGER K, KNOOP C AND HÖCKEL M. (1991). Oxygenation of human tumors: evaluation of tissue oxygen distribution in breast cancers by computerized $O_2$ tension measurements. *Cancer Res.*, **51**, 3316–3322.

WANG J, BIEDERMANN KA AND BROWN JM. (1992). Repair of DNA and chromosome breaks in cells exposed to SR 4233 under hypoxia or to ionizing radiation. *Cancer Res.*, **52**, 4473–4477.

WILSON WR AND DENNY WA. (1992). DNA-binding nitroheterocycles as hypoxia-selective cytotoxins. In *Radiation Research: A Twentieth-Century Perspective*, Dewey WC, Edington M, Fry RJM, Hall EJ and Whitmore GF (eds) pp. 796–801. Academic Press: New York.

WORKMAN P AND STRATFORD IJ. (1993). The experimental development of bioreductive drugs and their role in cancer therapy. *Cancer Metastasis Rev.*, **12**, 73–82.

ZEMAN EM AND BROWN JM. (1989). Pre- and post-irradiation radiosensitization by SR 4233. *Int. J. Radiat. Oncol. Biol. Phys.*, **16**, 967–971.

ZEMAN EM, BROWN JM, LEMMON MJ, HIRST VK AND LEE WW. (1986). SR-4233: a new bioreductive agent with high selective toxicity for hypoxic mammalian cells. *Int. J. Radiat. Oncol. Biol. Phys.*, **12**, 1239–1242.

ZEMAN EM, HIRST VK, LEMMON MJ AND BROWN JM. (1988). Enhancement of radiation-induced tumor cell killing by the hypoxic cell toxin SR 4233. *Radiother. Oncol.*, **12**, 209–218.

ZHANG H, KOCH CJ, WALLEN CA AND WHEELER KT. (1995). Radiation-induced DNA damage in tumors and normal tissues. III. Oxygen dependence of the formation of strand breaks and DNA-protein crosslinks. *Radiat. Res.*, **142**, 163–168.